# Supplementary material for: Pharmacological inhibition of host pathways enhances macrophage killing of intracellular bacterial pathogens
Source: Microbiol Spectr. 2025 Dec 3;14(1):e02163-25. doi: 10.1128/spectrum.02163-25 (PMC12772234; doi:10.1128/spectrum.02163-25)
Supplement: Supplemental figures — Figures S1 to S4. [file spectrum.02163-25-s0001.docx]

**Supplementary Figures**

**Figure S1**: **ScPPX, Rapamycin (mTOR inhibitor), or FPSZM1 (RAGE inhibitor) potentiates the ability of macrophages to kill ingested *Mtb.*** Viable ingested *Mtb* in macrophages with GM-CSF, in the absence (Control) or presence of 10 µg/ml ScPPX or 1000 nM of the indicated inhibitor, was determined as colony-forming units (CFU) at 4 hours (A) or 48 hours (B and C) after ingestion. For each experiment with each donor, CFU in the control was considered 100%. All values are mean ± SEM of six (three female and three male donors) independent experiments. Male data points are shown in blue and female data points are shown in red. For each bar, there was no significant difference between male and female (unpaired t-test). * p < 0.05 compared to control (one-way ANOVA, Dunnett's test).

**Figure S2**: **ScPPX or inhibitors do not significantly affect metabolic activity of infected macrophages or growth of bacteria.** (A – C) Uninfected macrophages or *Mtb*, *Legionella*, or *Listeria* infected macrophages in the absence or presence of 10 µg/ml ScPPX or the indicated inhibitor for 24 hours were incubated with Deep Blue Cell Viability resazurin dye for 12 hours, and fluorescence was measured. For each experiment with each donor, the average of the metabolic activity of uninfected macrophages was considered 100%. (D – F) *Mtb*, *Legionella*, or *Listeria* cultures were grown for 6 days (*Mtb*), 144 hours (Legionella), or 48 hours (*Listeria*) in the absence (Control) or presence of 1000 nM of the indicated inhibitor. The OD_600_ was measured daily. Values are mean ± SEM of six (three females and three males) (A and B), five (two female and three male) (C), or mean ± SD of six (D – F) independent experiments. For A – C, male data points are shown in blue and female data points are shown in red. For each bar in A-C, there was no significant difference between male and female (unpaired t-test).

**Figure S3: FPSZM1 prevents polyP-mediated inhibition of phagosome acidification in M-CSF macrophages.** (A) Human macrophages (Mφ) with GM-CSF were incubated without (top) or with yeast (bottom) for 1 hour, fixed, and fluorescence images were taken as in Figures 2 D and H. Differential interference contrast (DIC) images are at the left, DIC merged with fluorescence are in the middle, and fluorescence images of macrophages without (top) and with yeasts (bottom) are at the right. Bar is 20 µm. Images are representative of five independent experiments (two female and three male). (B - I) Human macrophages with GM-CSF (B – E) or M-CSF (F – I) were incubated with yeast, in the absence (Control) or presence of 15 µg/ml polyP (+ polyP) without (No drug) or with 1000 nM of the indicated inhibitor for 1 hour, fixed, fluorescence images were taken, and fluorescence intensities of yeast, percent of macrophages with yeasts, and number of yeasts per macrophages were determined as in Figure 2 D – K. DIC merged with fluorescence are at the left, and fluorescence images are at the right for each treatment condition. Bars are 20 µm. Images are representative of five independent experiments (two female and three male). Male data points are shown in blue and female data points are shown in red. For each bar, there was no significant difference between male and female (unpaired t-test). All values are mean ± SEM of five (two female and three male) independent experiments. *p < 0.05; **p < 0.01; ***p < 0.001 (one-way ANOVA with Dunnett's test).
